# Standardized *Centella asiatica* extract ECa 233 alleviates pain hypersensitivity by modulating P2X3 in trigeminal neuropathic pain

**DOI:** 10.1590/1678-7757-2023-0337

**Published:** 2024-01-05

**Authors:** Aree Wanasuntronwong, Supassanan Kaewsrisung, Nisanat Lakkhanachatpan, Rittinarong Meepong, Tawepong Arayapisit, Mayuree Tantisira

**Affiliations:** 1 Mahidol University Faculty of Dentistry Department of Oral Biology Bangkok Thailand Mahidol University, Faculty of Dentistry, Department of Oral Biology, Bangkok 10400, Thailand.; 2 Burapha University Faculty of Pharmaceutical Sciences Chonburi Thailand Burapha University, Faculty of Pharmaceutical Sciences, Chonburi 20131, Thailand.; 3 Mahidol University Faculty of Dentistry Department of Anatomy Bangkok Thailand Mahidol University, Faculty of Dentistry, Department of Anatomy, Bangkok 10400, Thailand.

**Keywords:** ECa 233, Infraorbital nerve chronic constriction, P2X3, NaV1, 7, c-fos

## Abstract

During oral surgery and temporomandibular joint repositioning, pain hypersensitivity often occurs due to irritation or inflammation of the nerve endings in the orofacial region.

**Objective:**

This study aimed to investigate the effects of ECa 233, a *Centella asiatica*–standardized extract, on the development of mechanical hyperalgesia and allodynia induced by chronic constriction injury of the infraorbital nerve in mice.

**Methodology:**

The right infraorbital nerves of the mice were ligated. Oral carbamazepine (20 mg/kg) or ECa 233 (30, 100, or 300 mg/kg) was administered daily for 21 days. Von Frey and air-puff tests were performed on both sides of the whisker pad on days 0, 7, 14, and 21. Thereafter, the expression of purinergic receptor subtype 3 (P2X3) and voltage-gated sodium channel 1.7 (NaV1.7), a transmembrane protein, in the trigeminal ganglion and c-fos immunoreactivity-positive neurons in the trigeminal nucleus caudalis was assessed.

**Results:**

After 21 days of infraorbital nerve ligation, the mice showed allodynia- and hyperalgesia-like behavior, P2X3 and NaV1.7 were upregulated in the trigeminal ganglion, and nociceptive activity increased in the trigeminal nucleus caudalis. However, the oral administration of carbamazepine (20 mg/kg), ECa 233 (100 mg/kg), or ECa 233 (300 mg/kg) mitigated these effects. Nevertheless, ECa 233 failed to affect NaV1.7 protein expression.

**Conclusion:**

Carbamazepine and ECa 233 can prevent pain hypersensitivity in mice. Considering the side effects of the long-term use of carbamazepine, ECa 233 monotherapy or combined ECa 233 and carbamazepine therapy can be used as an alternative for regulating the development of hypersensitivity in trigeminal pain. However, further detailed clinical studies should be conducted to provide comprehensive information on the use of ECa 233.

## Introduction

Trigeminal neuropathic pain consists of persistent and often debilitating discomfort due to damage or dysfunction of the trigeminal nerve. This nerve transmits sensory signals from the orofacial region to the brain.^1^ This type of pain typically manifests itself as sharp, burning, or shooting sensations in the face and tends to be long-lasting. Hyperalgesia and allodynia configure two common features associated with trigeminal neuropathic pain. Hyperalgesia stems from damage or irritation to the trigeminal nerve, which leads to its malfunction and increases its sensitivity to pain signals. Consequently, this increased sensitivity can increase pain perception.^2^ Additionally, nerve damage may induce the interaction of the affected nerve with neighboring sensory nerves or adjacent nerve pathways, which can cause allodynia, a condition in which normally non-painful sensations are perceived as painful.^2^ Inflammatory cytokines often mediate the underlying pathophysiological mechanism of pain hypersensitivity, which can directly sensitize nerve endings in the affected area by interacting with receptors on nerve fibers. This sensitization reduces the pain threshold and increases responsiveness to pain signals, making even mild stimuli painful. A significant factor in this process is the P2X3 receptor, a purinergic receptor activated by the signaling molecule adenosine triphosphate (ATP).^3^ The activation of P2X3 receptors sensitizes sensory nerve fibers, increasing their responsiveness to pain signals and leading to allodynia.^4^ Notably, nerve injury cases specifically show P2X3 receptor overexpression.^5^ Nav1.7 is a specific type of voltage-gated sodium channel (known as the Nav1.7 channel) that plays a crucial role in pain perception and hypersensitivity. Nav1.7 channels primarily occur in peripheral sensory neurons, especially in small-diameter nociceptive neurons.^6^ Mutations that enhance the activity of Nav1.7 channels can increase neuron excitability, raising their readiness and frequency of responses to painful stimuli. This increased excitability contributes to pain hypersensitivity.^7^

Although carbamazepine (CBZ) is primarily prescribed to manage epilepsy and specific types of seizures, it can also treat certain neuropathic pain conditions.^8^ The mechanism of action of CBZ involves stabilizing nerve cell membranes by blocking voltage-gated sodium channels, including Nav1.7 channels.^8,9^ These sodium channels are essential to generate and transmit electrical signals in nerve cells. By inhibiting them, CBZ can reduce the abnormal firing of neurons associated with neuropathic pain.^9^ Despite its effectiveness, CBZ can cause adverse reactions such as drowsiness, dizziness, and nausea, which thus leads to treatment withdrawal in about 20% of cases. Furthermore, CBZ is associated with life-threatening reactions, including Stevens-Johnson syndrome, agranulocytosis, aplastic anemia, and drug-drug interactions by hepatic cytochrome P450 induction.^10^ Some herbal products are available online as an alternative treatment for pain hypersensitivity, but very few scientific reports support their safety and effectiveness.

The standardized *Centella asiatica* extract ECa 233, which contains at least 80% triterpenoid glycosides and maintains the madecassoside-to-asiaticoside ratio at 1.50:0.50,^11^ has a safety profile for acute and sub-chronic toxicity.^12^ ECa 233 exerts antinociceptive effect in the trigeminal neuropathic pain by modulating the expression of calcitonin gene-related peptide (CGRP) on the trigeminal ganglion.^13^ It also helps to manage temporomandibular joint osteoarthritis pain induced by complete Freund’s adjuvant via the transient receptor potential vanilloid 1 and the acid sensing ion channel subunit 3 protein expression on the articular surface of condylar head.^14^ A previous report showed that madecassoside, a major active component of ECa 233, can prevent inflammation by inhibiting the expression of cyclooxygenase-1, cyclooxygenase-2, and prostaglandin E2 in an arthritis mouse model.^15^ After three weeks of trigeminal neuropathic pain induction, ECa 233 exerts antinociceptive effects by downregulating CGRP expression in the trigeminal ganglion. However, the effectiveness of ECa 233 in mitigating pain development during chronic constriction injury by P2X3 and NaV1.7 peripheral sensitization is yet to be evaluated.

Our study aimed to investigate the effect of ECa 233 on the development of pain hypersensitivity induced by chronic constriction injury in mice. It also examined the expression of P2X3 and NaV1.7 in the trigeminal ganglion (TG) and c-fos (a molecular marker of neuronal activity) in the trigeminal nucleus caudalis (TNC).

## Methodology

### Study groups and experimental design

The procedures for housing and treating animals in this study were authorized by the Animal Care and Use Committee of the Faculty of Tropical Medicine at Mahidol University (FTM-ACUC 004/2017) and were in accordance with the ethical standards outlined by the International Association for the Study of Pain for research involving conscious animals.^16^

**Figure 1:**
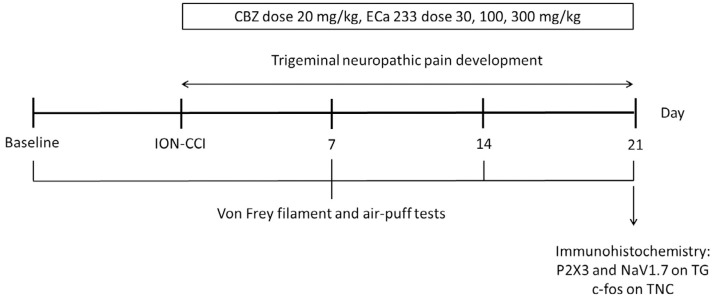
Timeline of the experimental design. CBZ; Carbamazepine, ECa 233; a standardized Centella asiatica extract, ION-CCI; infraorbital nerve chronic constriction injury, P2X3; purinergic receptor subtype 3, NaV1.7; sodium voltage channel 1.7, TG; trigeminal ganglion, TNC; trigeminal nucleus caudalis

Our study involved 60 six-week-old male Institute of Cancer Research mice, each weighing 25-30 g. These mice were obtained from Nomura Siam International (Bangkok, Thailand). Sample size was set at 80% statistical power (1-β) with a 95% confidence interval (α = 0.05).^13^ Mice were randomly assigned to one of the following six groups (8-10 animals per group): sham + 0.5% carboxymethyl cellulose (CMC), infraorbital nerve chronic constriction injury (ION-CCI) + 0.5% CMC, ION-CCI + CBZ 20 mg/kg, ION-CCI + ECa 233 30 mg/kg, ION-CCI + ECa 233 100 mg/kg, and ION-CCI + ECa 300 mg/kg. In total, five mice were kept in each cage under controlled environmental conditions (22°C ± 2°C regulated temperature and 45% ± 15% humidity) and a 12-hour light/dark cycle. Von Frey filaments and air-puff tests were conducted before performing the procedure to induce ION-CCI and seven, 14, and 21 days after ION-CCI. The mice were humanely euthanized one hour after the performance of behavioral tests on day 21 and immunohistochemical studies were carried out to assess the expression of P2X3 and NaV1.7 in TG and c-fos in TNC (Figure 1).

### Chemicals and test compounds

ECa 233, a standardized *Centella asiatica* extract, was provided by Siam Herbal Innovation (Lot No. MRA051401). The sham and ION-CCI groups were orally administered 0.3 mL of a 0.5% CMC solution; the CBZ-treated groups (C4024, sigma), 0.3 mL of CBZ (dissolved in 0.5% CMC) at a concentration of 20 mg/kg daily; and the ECa 233-treated groups, 0.3 mL dose of ECa 233 (dissolved in 0.5% CMC) at 30, 100, or 300 mg/kg concentrations via oral gavage daily for 21 days following the ION-CCI procedure. All test substances were administered one hour before nocifensive behaviors were assessed.

### Chronic constriction injury of the infraorbital nerve (ION-CCI)

The ION-CCI procedure involved anesthetizing the mice with isoflurane. Isoflurane concentration ranged from 4 to 5% during induction and was maintained at 2-2.5% during surgical procedures. Aseptic conditions were maintained during intraoral surgery. A 4-mm-long incision was made in the anterior-posterior orientation along the gingivobuccal margin behind the first molar. The right infraorbital nerve was loosely tied using a 4-0 silk thread. Caution was taken to prevent excessive nerve compression, which could inhibit the development of allodynia in rats.^17^ Overall, two ligatures were placed with an approximate space of 1 mm between them. In the sham operation, the infraorbital nerve was exposed but left untouched. Following nerve ligation, the mice were closely monitored until they had fully recovered.

### Assessment of nocifensive behaviors

All behavioral tests were conducted from 07:00 to 12:00 am. The von Frey and air-puff tests were performed before and the development of ION-CCI and seven, 14, and 21 days after it.

In the von Frey test, each mouse was placed and acclimatized for at least 15 min in a restraining device, and free movement of the head and front paws were allowed. Based on our previous study,^18^ von Frey filaments (with a sub-threshold stimulus of 0.008 g and a suprathreshold stimulus of 0.07 g; Ugo Basile SRL, Gemonia VA, Italy) were applied at a 90° angle to the whisker pad skin until slightly bent. Each side of the whisker pad was stimulated 12 times.

In the air-puff test, a constant pressure of 10 psi was applied 12 times on each side of the whisker pad skin. Air-puff pressure was applied using a 10-cm-long metal tube 1 cm from the skin at a 90° angle. Animal behavior was video-recorded and scored as follows: 0, no response; 0.25, a clear withdrawal of the head from the stimulus or the presence of grabbing/biting behavior in response to the stimulus; 1, unilateral or bilateral forepaw swipe across the face; and 1.5, continuous forepaw swipes (at least thrice) across the face. This scoring system was based on Krzyzanowska, et al.^19^ (2011). Scores obtained in the 12 rounds of testing were added to obtain the total score.

### Tissue preparation and immunohistochemistry

Immunohistochemistry was used to investigate P2X3 and NaV1.7 expressions in TG and c-fos expression in TNC. At the end of the experiment, the mice were deeply anesthetized and subjected to transcardial perfusion using phosphate buffered saline (PBS, 0.1 M, pH 7.4). TG and TNC were removed and immersed in 4% paraformaldehyde. TG was infiltrated with paraffin wax and embedded for microtome sectioning. The thickness of TG sections totaled 4 μm. TG sections were deparaffinized and warmed with antigen retrieval solution (Tris-EDTA, pH 9.0). Endogenous peroxidase activity was inhibited by exposing the samples to 3% H_2_O_2_ for 10 min. Nonspecific staining was blocked by exposing the samples to an antibody diluent (Agilent Dako) for 1 h. Sections were incubated with mouse monoclonal anti-P2X3 (sc-390572; Santa Cruz Biotech, Dallas, TX, USA) or rabbit monoclonal anti-NaV1.7 antibodies (AB5390; Merck KGaA, Darmstadt, Germany) at 4°C overnight. They were then treated with goat anti-rabbit IgG H&L (HRP) (Abcam) for 1 h at 25°C ± 1°C. The samples were stained with 3,3’-diaminobenzidine (DAB, Agilent Dako) for 5 min and counterstained with hematoxylin. After staining, the sections were examined under a light microscope. Overall, three sections from each mouse were selected to quantify immunopositive neurons. The average ratio of immunopositive neurons to total neurons in each animal was calculated.

TNC was immersed in 30% sucrose until the tissues sank. They were then cut using a cryostat at −20°C to obtain 30-μm thick sections. The free-floating method was used to stain these sections. Endogenous peroxidase activity was inhibited by exposing the samples to 3% H_2_O_2_ for 5 min, and nonspecific staining was blocked by exposing the samples to 5% bovine serum albumin for 1 h. Sections were incubated with rabbit polyclonal anti-c-fos (1: 500 dilution, Abcam) at 4°C overnight. Then, they were treated with goat anti-rabbit IgG H&L (HRP) (Abcam) for 1 h at 25°C ± 1°C. DAB (Agilent Dako) was applied for 5 min. Next, the sections were mounted onto slides and dehydrated. Immunostained sections were observed under a light microscope. The number of immunopositive neurons in laminas I and II of the TNC was counted (three sections per mouse). The average number of c-fos-immunopositive neurons was calculated for each mouse.

### Data analysis

All results are expressed as mean ± SEM. Two-way repeated-measure analysis of variance (ANOVA), followed by the Tukey’s test, was used to analyze nocifensive behavior scores for all 12 times. One-way ANOVA, followed by the Duncan T3 Alpha’s multiple comparisons test, was used to analyze P2X3-, NaV1.7-, and c-fos IR-positive neurons. A p-value of <0.05 was considered statistically significant.

## Results

After 21 days of ION-CCI, mice in the sham, ION-CCI, ION-CCI receiving CBZ 20 mg, ION-CCI receiving ECa 233 30 mg, ION-CCI receiving ECa 233 100 mg, and ION-CCI receiving ECa 233 300 mg groups showed no weight difference.

### Effects of ECa 233 on nocifensive behaviors after ION-CCI

#### Air-puff stimulation

**Figure 2:**
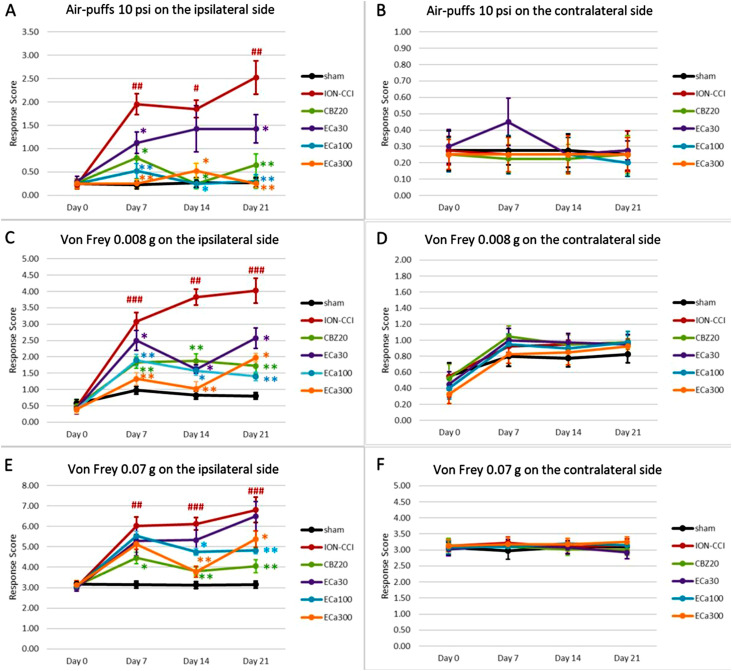
Graphs show the protective effects of oral Carbamazepine (20 mg/kg) and ECa233 (30, 100, and 300 mg/kg) against the development of the nocifensive behavior response induced by 10 psi air-puff and 0.008 and 0.07 g of von Frey filament on the ipsilateral (A, C, E) and contralateral (B, D, F) sides of winkle pads mice (mean ± SEM for 10 mice). #p<0.05, ##p<0.01, and ###p<0.001 compared with the sham group. *p<0.05 and **p<0.01 compared with the ION-CCI group. Two-way ANOVA with Tukey’s multiple comparisons test. CBZ; carbamazepine, ION-ICC; chronic constriction injury of the infraorbital nerve, ECa233; Centella asiatica-standardized extract

Ipsilateral response scores in the ION-CCI group were significantly higher than those in the sham group from day 7 to day 21 after ION-CCI [F(15, 216) = 5.252, p≤0.01 (day 7), p≤0.05 (day 14) and p≤0.01 (day 21)]. Day 21 showed the maximum score. Ipsilateral response scores significantly decreased in mice treated with CBZ (20 mg/kg) on days 7, 14, and 21 [F(15, 216) = 5.252, p≤0.05 (day 7 and day 14), p=0.01 (day 21); n=10]. Mice that received ECa 233 (30 mg/kg) had significantly decreased ipsilateral response scores on days 7 and 21 [F(25, 210) = 5.252, p=0.05 (days 7 and 21); n=10]. Moreover, mice treated with ECa 233 (100 and 300 mg/kg) showed a significant decrease in ipsilateral response scores from day 7 to day 21 [F (25, 210) = 5.252, p=0.01 (day 7), p≤0.05 (day 14) and p≤0.01 (day 21); n=10] (Figure 2A). However, testing groups showed no notable differences in contralateral response scores [F(15, 216) = 0.142; n=10] (Figure 2B).

#### Stimulation using 0.008 g von Frey filament

Ipsilateral response scores in the ION-CCI group were significantly higher than those in the sham group from day 7 to day 21 after ION-CCI [F(15, 216) = 8.228, p≤0.001 (day 7 and day 21) and p≤0.01 (day 14); n=10]. Day 21 showed the maximum score. Ipsilateral response scores were significantly reduced in mice treated with CBZ (20 mg/kg) from day 7 to day 21 [F(15, 216) = 8.228, p≤0.01; n=10]. Mice treated with ECa 233 (30 mg/kg) showed significantly decreased ipsilateral response scores from day 7 to 21 [F(25, 210) = 8.228, p≤0.05; n=10]. Mice treated with ECa 233 (100 mg/kg) showed significantly decreased ipsilateral response scores from day 7 to day 21 [F(25, 210) = 8.228, p≤0.01 (day 7 and day 21) and p≤0.05 (day 14); n=10]. Similarly, mice administered with 300 mg/kg ECa 233 had a significant decrease in ipsilateral response scores from day 7 to day 21 [F(25, 210) = 8.228, p≤0.01 (day 7 and day 14), p≤0.05 (day 21); n=10] (Figure 2C). However, testing groups showed no notable differences in contralateral response scores [F(15, 216) = 0.236; n=10] (Figure 2D).

#### 0.07 g von Frey filament stimulation

Ipsilateral response scores in the ION-CCI group were significantly higher than those in the sham group from day 7 to day 21 after ION-CCI [F(15, 216) = 3.716, p≤0.01 (day 7) and p≤0.001 (day 14 and day 21); n=10]. Day 21 showed the highest score. Ipsilateral response scores were significantly reduced in mice treated with CBZ (20 mg/kg) from day 7 to day 21 [F(15, 216) = 3.718, p≤0.05 (day 7) and p≤0.01 (days 14 and 21); n=10]. Similarly, mice treated with ECa 233 (100 mg/kg) showed a significant decrease in ipsilateral response scores on days 14 and 21 [F(25, 210) = 3.718, p≤0.05 (day 14) and p≤0.01 (day 21); n=10]. ECa 233 (300 mg/kg) showed a significant decrease in ipsilateral response scores on days 14 and 21 [F(25, 210) = 3.718, p≤0.01 (day 14) and p≤0.05 (day 21); n=10] (Figure 2E). The administration of ECa 30 failed to significantly affect nocifensive behavior in mice subjected to stimulation with a 0.07 g von Frey filament [F(15, 216) = 3.718; n=10]. As observed in previous experiments, testing groups showed no significant differences in contralateral response scores [F(15, 216) = 0.236; n=10] (Figure 2F).

## Effects of ECa 233 on P2X3 and NaV1.7 expression in the TG

**Figure 3:**
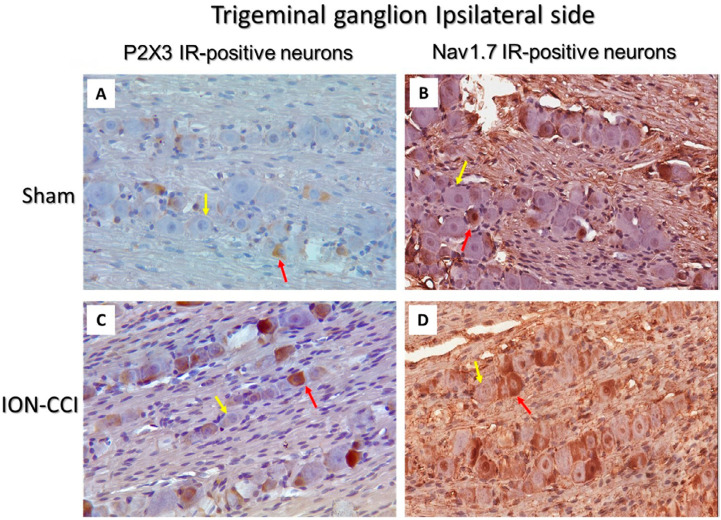
Photos show the characteristic of P2X3 immunoreactivity (IR)-positive neurons on the ipsilateral trigeminal ganglion of the sham and ION-CCI groups after three weeks of intraorbital nerve ligation (40× microscope). The red arrow indicates P2X3 or NaV1.7 IR-positive neurons and the yellow arrow, the negative trigeminal neuron. ION-CCI; infraorbital nerve chronic constriction injury, P2X3; purinergic receptor subtype 3, NaV1.7; sodium voltage channel 1.7

**Figure 4:**
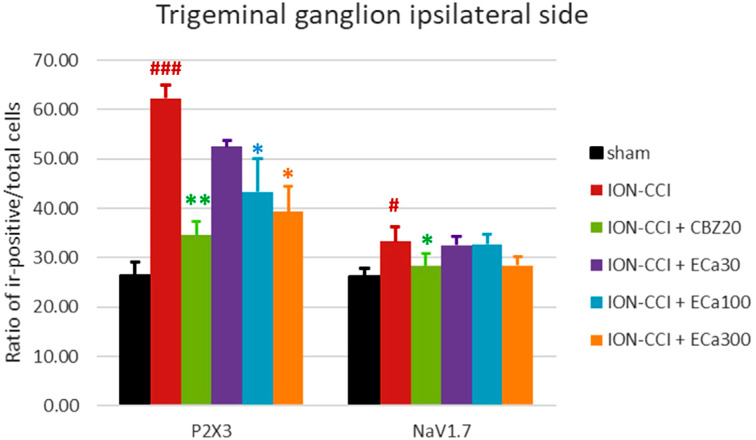
The graph represents the protective effects of oral CBZ (20 mg/kg) and ECa 233 (30, 100, and 300 mg/kg) on ipsilateral P2X3- and NaV1.7 expression in the trigeminal ganglion after three weeks of intraorbital nerve ligation in mice (mean ± SEM for 8 mice). #p<0.05 and ###p<0.001 compared with the sham group. *p<0.05 and **p<0.01 compared with the ION-CCI group. One-way ANOVA with Duncan T3 Alpha’s multiple comparisons test. CBZ; carbamazepine, ION-ICC; chronic constriction injury of the infraorbital nerve, ECa 233; Centella asiatica–standardized extract

Figure 3 illustrates the characteristics of TG neurons, specifically P2X3- and NaV1.7 immunoreactive (IR) positive neurons. The total number of ipsilateral TG used to calculate the ratio of P2X3- and NaV1.7 IR-positive neurons showed no significant differences across groups [F(5,42) = 0.181, p=0.962; n=8] (Table 1). On day 21, ION-CCI mice showed significantly higher P2X3 IR-positive neurons in the ipsilateral TG than the sham-operated mice. However, ION-CCI mice treated with CBZ (20 mg/kg) and ECa 233 (100 and 300 mg/kg) showed a reverse effect, which was absent in ION-CCI mice treated with ECa 233 (30 mg/kg) [F(5,42) = 10.418, p≤0.001; n=8] (Figure 4). Similarly, ION-CCI mice showed significantly higher NaV1.7 IR-positive neurons in the ipsilateral TG than the sham-operated mice. However, this upregulation of NaV1.7 IR-positive neurons was reversed in ION-CCI mice treated with CBZ (20 mg/kg). Interestingly, all doses of ECa 233 failed to inhibit the upregulation of NaV1.7 IR-positive neurons following the ION-CCI operation [F(5,42) = 1.851, p=0.124; n=8] (Figure 4).

**Table 1:** Total number of ipsilateral trigeminal ganglion neurons to calculate the ratio of P2X3- and NaV1.7 IR-positive neurons

	Neuron (counts)
Group	Ipsilateral side
sham	349.75 ± 31.33
ION-CCI	373.12 ± 27.55
ION-CCI + CBZ20	345.75 ± 31.48
ION-CCI + ECa30	358.65 ± 17.78
ION-CCI + ECa100	369.75 ± 28.64
ION-CCI + ECa300	454.50 ± 9.48

P2X3, a purinergic receptor subtype 3; NaV1.7, voltage-gated sodium channel subtype 1.7; TG, trigeminal ganglion; ION-ICC, chronic constriction injury of the infraorbital nerve; CBZ, carbamazepine; ECa233, Centella asiatica-standardized extract Data are expressed as the mean ± standard error of mean (mean ± SEM; n = 8).

## Effects of ECa 233 on c-fos expression in the TNC

**Figure 5:**
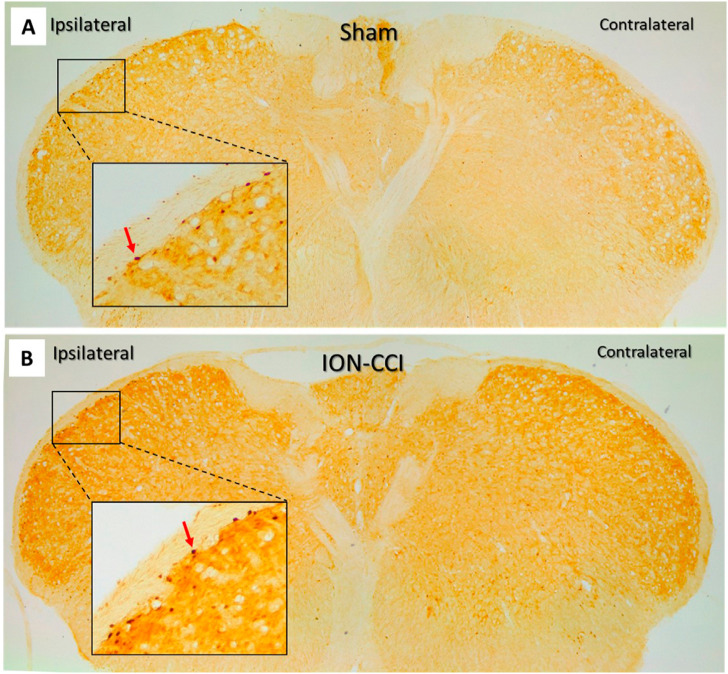
Photos show the characteristics of c-fos-immunoreactivity–positive neurons on the trigeminal nucleus caudalis of the sham and ION-CCI groups after three weeks of intraorbital nerve ligation. (4× and 10× inset). The red arrow at 10× inset indicates c-fos IR-positive cells. ION-CCI, chronic constriction injury of the infraorbital nerve

**Figure 6:**
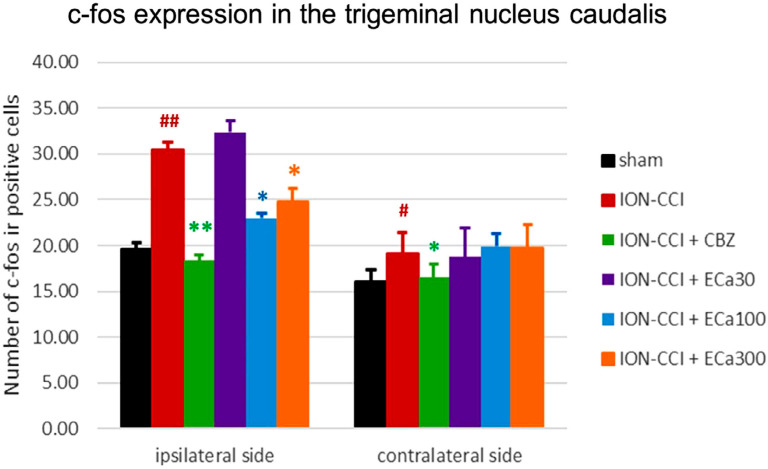
The graph represents the protective effects of oral CBZ (20 mg/kg) and ECa 233 (30, 100, and 300 mg/kg) on c-fos expression in the trigeminal nucleus caudalis after three weeks of intraorbital nerve ligation in mice (mean ± SEM for 8 mice). #p<0.05 and ###p<0.001 compared with the sham group. *p<0.05 and **p<0.01 compared with the ION-CCI group. One-way ANOVA with Duncan T3 Alpha’s multiple comparisons test. CBZ; carbamazepine, ION-ICC; chronic constriction injury of the infraorbital nerve, ECa233; Centella asiatica–standardized extract

Figure 5 describes the characteristics of c-fos IR-positive cells. On day 21, the expression of c-fos IR-positive cells in the ipsilateral TNC was significantly higher in the ION-CCI group than in the sham group. Treating the mice with CBZ (20 mg/kg) and ECa 233 (100 and 300 mg/kg) mitigated this increase in c-fos IR positive cells but ECa 233 (30 mg/kg) failed to do so [F(5,42) = 36.208, p≤0.001; n=8]. On the contralateral side, the ION-CCI group showed an increased c-fos IR-positive cells when compared with the sham-operated group (Figure 6).

## Discussion

This study primarily aimed to examine the impact of a standardized extract derived from *Centella asiatica* (ECa 233) on the development of trigeminal neuropathic pain in mice with nerve injuries. It assessed the presence and progression of trigeminal neuropathic pain by monitoring hyperalgesia and allodynia at various time points. Results showed a gradual increase in nocifensive behaviors over a 21-day period after stimulation with von Frey filaments (indicating the presence of hyperalgesia)^19^ and air-puff stimuli, indicating allodynia^19^ in mice with ION-CCI. This outcome confirmed the successful establishment of trigeminal neuropathic pain in this experimental model.

The administration of CBZ and ECa 233 can mitigate the development of hyperalgesia and allodynia due to ION-CCI. Although ECa 233 effectively prevented hyperalgesia and allodynia development, a low dose of ECa 233 failed to alleviate hyperalgesia. This observation suggests the possibility that distinct mechanisms are involved in the development of hyperalgesia and allodynia. Notably, the sprouting or regrowth of nerve fibers, especially A-fibers, after nerve injury plays a pivotal role in the occurrence of allodynia.^20^ Due to the difference in the effects of low and high doses of ECa 233, we postulate that it may modulate the mechanism inducing allodynia more than hyperalgesia. Peripheral sensitization is crucial in the progression of hyperalgesia and allodynia.^21^ This process involves the release of inflammatory mediators, such as prostaglandins, bradykinin, and histamine, which usually increase the sensitivity to stimuli and hypersensitivity in individuals.^21^ Concurrently, alterations in the ion channels in nerve cell membranes render them more permeable to sodium ions. This change in ion channel function increases excitability in nerve endings and leads to exaggerated responses to various stimuli.^21^

This study found the expression of P2X3 and NaV1.7 within the TG, which played a key role in the peripheral sensitization mechanism. A previous study showed that P2X3 can mitigate the development of hyperalgesia due to carrageenan^22^ and allodynia by resiniferatoxin.^23^ Both CBZ and ECa 233 showed the capacity to reduce the upregulation of P2X3 in the TG, indicating their protective effect against inflammatory modulation-induced peripheral sensitization. Koizumi et al. showed that CBZ can inhibit the upregulation of P2X3 in a model involving compression of the trigeminal nerve root.^24^ Their research indicated that this inhibition involves the signaling of TNFα released from activated macrophages.^25^ Alternatively, ECa 233 can suppress proinflammatory cytokine responses in macrophages, including TNF-α, PGE2, and IL-1β,^26^ and exert antioxidant properties in a chronic stress mice model.^11^ These mechanisms collectively suggest that ECa 233 can effectively mitigate the upregulation of P2X3 in ION-CCI. In addition to the observed overexpression of NaV1.7 during the development of trigeminal neuropathic pain, it is noteworthy that CBZ can counteract this effect, unlike ECa 233. CBZ is renowned for its ability to inhibit sodium voltage channels (including NaV1.7) and modulate the γ-aminobutyric acid subtype A receptor by interacting with its alpha 1, beta 2, and gamma 2 subunits.^9^ The protective mechanism, associated with CBZ, seems to be involved in the upregulation of the activating transcription factor 4 (ATF-4).^27^ Notably, Li, et al.^28^ (2020) showed that increased ATF-4 levels can reduce the expression of sodium voltage channels in a neuropathic hypersensitivity model. Conversely, madecassic acid, a bioactive compound in *Centella asiatica*, fails to impact ATF-4.^29^ This observation suggests the potential existence of distinct mechanisms governing the effects of ECa 233 and CBZ on the expression of NaV1.7 channels.

C-fos is a common marker for the identification and quantification of neuronal activation within the TNC. Activated TNC neurons in response to pain often express c-fos as part of their immediate early gene response.^30^ The response to persistent pain stimuli in mice with ION-CCI that received ECa 233 and CBZ ultimately consisted of the summation of peripheral sensitization via P2X3 and NaV1.7. Both ECa 233 and CBZ effectively mitigated the expression of c-fos on the ipsilateral side, but a low dose of ECa 233 failed to replicate this effect. This finding corresponded to the observed nocifensive behavior and the expression of P2X3/NaV1.7 in the TG. Interestingly, the contralateral side of the TNC showed increased c-fos expression in the CCI group. This phenomenon may be attributed to the mirror-image pain response, which is potentially linked to the widespread presence of inflammatory mediators along the corresponding dermatomal levels.^18^

Considering that the antineuropathic pain effect of ECa 233 is comparable to that of CBZ, it can be inferred that ECa 233 metabolism fails to involve the cytochrome P450. Hence, ECa 233 could be safer than CBZ. In animal acute and subacute toxicity tests, no mice that received a single dose of ECa 233 (10 g/kg) died. In a previous study, rats receiving Eca 233 up to 1000 mg/kg for 90 days showed no major adverse effects.^12^ The pharmacokinetics of ECa 233 in healthy volunteers has recently been studied.^31^ The proof of efficacy in patients with trigeminal neuropathic pain could further develop ECa 233 as a possible alternative treatment agent for trigeminal neuropathic pain, probably as an adjuvant to CBZ.

## Conclusion

This study showed the effect of ECa 233 on the development of pain hypersensitivity during chronic constriction injury-induced neuropathic pain, with reference to carbamazepine. The underlying mechanism of ECa 233 may differ from that of CBZ. Hence, their synergistic effect should be investigated. Furthermore, as ECa 233 is safe and its pharmacokinetic properties in humans are known, clinical research should evaluate the possibility of using ECa 233 as a food supplement in patients with trigeminal neuropathic pain.

## Data Availability

The datasets generated and/or analyzed in this study are available from the corresponding author upon reasonable request.
